# Chloride content of solutions used for regional citrate anticoagulation might be responsible for blunting correction of metabolic acidosis during continuous veno-venous hemofiltration

**DOI:** 10.1186/s12882-016-0334-3

**Published:** 2016-08-26

**Authors:** Rita Jacobs, Patrick M. Honore, Marc Diltoer, Herbert D. Spapen

**Affiliations:** Intensive Care Department, Universitair Ziekenhuis Brussel, Vrije Universiteit Brussel, 1090 Brussels, Belgium

**Keywords:** Citrate, Anticoagulation, Continuous veno-venous hemofiltration, Acid-base balance, Chloride, Acidosis, Alkalosis, Hyperchloremic acidosis, Strong ion gap, Stewart-Figge approach

## Abstract

**Background:**

Citrate, the currently preferred anticoagulant for continuous veno-venous hemofiltration (CVVH), may influence acid-base equilibrium.

**Methods:**

The effect of 2 different citrate solutions on acid-base status was assessed according to the Stewart-Figge approach in two consecutive cohorts of critically ill adult patients. The first group received Prismocitrate 10/2 (PC10/2; 10 mmol citrate/L). The next group was treated with Prismocitrate 18/0 (PC18; 18 mmol citrate/L). Both groups received bicarbonate-buffered fluids in post-dilution.

**Results:**

At similar citrate flow, the metabolic acidosis present at baseline in both groups was significantly attenuated in PC18 patients but persisted in PC10/2 patients after 24 h of treatment (median pH 7,42 vs 7,28; *p* = 0.0001). Acidosis in the PC10/2 group was associated with a decreased strong ion difference and an increased strong ion gap (respectively 43 vs. 51 mmol/L and 17 vs. 12 mmol/L, PC10/2 vs. PC18; both *p* = 0.001). Chloride flow was higher in PC10/2 than in PC18 subjects (25.9 vs 14.3 mmol/L blood; *p* < 0.05).

**Conclusion:**

Correction of acidosis was blunted in patients who received 10 mmol citrate/L as regional anticoagulation during CVVH. This could be explained by differences in chloride flow between the applied citrate solutions inducing hyperchloremic acidosis.

## Background

Continuous veno-venous hemofiltration (CVVH) under regional citrate anticoagulation (RCA) is increasingly used for treatment of acute kidney injury (AKI) in critically ill patients [1]. As a calcium-chelating agent, citrate effectively blocks coagulation activation in the extracorporeal circuit which enhances filter lifespan at low risk of bleeding [2]. However, citrate may cause adverse metabolic effects. Citrate metabolization produces bicarbonate, hence generating metabolic alkalosis. Conversely, in patients with decreased capacity to metabolize citrate (eg hepatic failure), accumulation can lead to high anion-gap metabolic acidosis [3–5]. Acid-base status is also affected by the amount of administered chloride and bicarbonate buffer, and occasionally by respiratory (over)compensation [6, 7]. Finally, when administered as a trisodium salt, excess citrate may induce hypernatremia [8].

Egi et al. were the first to document changes in acid-base balance in patients undergoing RCA-CVVH [9]. Since then, many attempts were undertaken to find the optimal citrate solution that allowed swift and effective control of acidosis whilst avoiding rebound alkalosis during continuous renal replacement therapy (CRRT) [10, 11]. Within this context, we studied the metabolic impact of two different commercially available citrate formulas (Prismocitrate 10/2 (PC10/2; 10 mmol citrate/L, Gambro-Hospal) and Prismocitrate 18/0 (PC18; 18 mmol citrate/L, Gambro-Hospal) in a cohort of critically ill patients with AKI undergoing RCA-CVVH. We based our metabolic approach on the Stewart-Figge method [12–15] to assess whether a difference existed in occurrence of metabolic acidosis between the two citrate protocols and to determine which factor(s) affected this metabolic disorder.

## Methods

The study was performed in the intensive care unit (ICU) of the University Hospital Brussels and conducted in compliance with the Helsinki Declaration. The Central Ethical Committee of the University Hospital approved the study protocol (BUN 143201318818). Due to its retrospective and observational before-after design, the need for informed consent was waived.

Patients were eligible when presenting AKI requiring RCA-CVVH treatment. Inclusion criteria were: age ≥ 18 years, presence of at least an AKI “RIFLE injury” score [16], and no contra-indication for RCA. Exclusion criteria were: patients already treated with CVVH during their ICU stay or receiving CVVH at time of enrolment, a high likelihood of dying within the first 24 h, impossibility to provide a correct vascular access, and Child-Pugh grade C liver cirrhosis. Baseline characteristics, including causes of AKI and incidence of shock are given in Table 1.

**Table 1:** Patient characteristics and causes of AKI

	Prismocitrate 10/2	Prismocitrate 18	*P* value
Number of patients (*n*)	28	31	
Age (years) (median [range])	73 (65–82)	68 (55–82)	0.298
Male gender (*n*, %)	17/28 (61 %)	21/31 (68 %)	0.598
Underlying disease/condition & cause of AKI (*n*, %)			
Sepsis	9/28 (32 %)	9/31 (29 %)	0.98
Post cardiac surgery	5/28 (18 %)	10/31(32 %)	0.33
Post general surgery	7/28 (25 %)	4/31(13 %)	0.39
Other	7/25 (25 %)	8/31(26 %)	0.90
APACHE II score (median [range])	32 (23–41)	27 (20–34)	0.109
Mechanical ventilation (days) (median [range])	11 (1–24)	14.6 (2–26)	0.167
Vasopressor requirement (*n*, %)	86 %	77 %	0.451

*Abbreviations*: *APACHE II* acute physiology and chronic health evaluation score II, *LOS* length of stay, *AKI* acute kidney injury

Patients were not randomized to receive either PC10/2 or PC18 but consecutively included within one treatment group. In fact, RCA with PC10/2 was initially applied in all patients on CVVH. At a given moment, we decided to replace PC10/2 by PC18. The reasons for changing the concentration of citrate were the followings: initially, all patients requiring CVVH in our ICU received PC10/2 for regional anticoagulation. To obtain better metabolic control (and especially better control of metabolic acidosis), we decided to replace PC10/2 (10 mmol citrate/L) by PC18 (18 mmol citrate/L). For practical reasons (storage capacity, short shelf-life of the citrate liquids, potential prescription errors), the hospital pharmacy did not make the two citrate solutions simultaneously available but delivered the PC18 solution after the PC10/2 stock was entirely consumed. Thus, a first group of patients received PC10/2 and, subsequently, a second group of patients was started on PC18. Regarding citrate dosage used during the study, these two dosage regimens (PC 18 & PC 10/2) fall within accepted dosing range for citrate anticoagulation. Solutions were not homemade but are CE-labelled and marketed by Gambro-Baxter. In- and exclusion criteria were identical for both study periods**.** CVVH was performed with the Prismaflex device (Gambro, Lund, Sweden) using an acrylonitrile 69 surface treated (AN69 ST) 150 membrane. Veno-venous access was obtained via a 13 F double-lumen polyurethane catheter (Joline, Swiss Confederation) inserted in the right internal jugular or a femoral vein. CVVH was delivered according to a dedicated protocol inspired by Tolwani et al. [17] and presented in detail previously [6]. This included standardized order sets and initial settings for all patients. Blood flow rate was set at 150 mL/min. Calcium chloride initially ran at 6 mL/h through a separate central venous line. Calcium infusion was titrated to maintain plasma ionized calcium levels between 1,0 and 1,2 mmol/L [6]. PC10/2 was delivered before the filter and started at a rate of 2200 mL/h. A bicarbonate-buffered solution (Prismasol 2) was infused in post-dilution, starting at 800 mL/h. In the PC18 group, citrate was delivered at a starting rate of 1500 mL/h and another bicarbonate buffer (Prismocal B22) in post-dilution at 400 mL/h. Detailed characteristics of the citrate and substitution fluids are shown in Table 2. Arterial blood gases, lactate, and serum electrolytes, including systemic and post-filter ionized calcium, were analyzed every 4 h. Acid-base status was evaluated with the Stewart-Figge method. This approach postulates that acid-base balance and pH depend on the difference between concentrations of strong cations and strong anions (ie the strong ion difference; SID), the PaCO_2_, and the total concentration of weak acids. It introduces the term “apparent strong ion difference” (SIDa) calculated as: ([Na^+^] + [K^+^] + [Mg^2+^] + [Ca^2+^]) - ([Cl^−^] - [lactate^−^]) (concentrations in mmol/L). The normal range for SIDa is approximately 40–44 mmol/L [18]. Since this equation does not account for weak acids (albumin, phosphate and CO_2_), the effective strong ion difference (SIDe) was calculated as (1000 × 2.46 × 10^−11^ × pCO_2_ / 10^-pH^) + [albumin] × (0.12 × pH - 0.631) + [phosphate] × (0.309 × pH - 0.469) (with pCO_2_ in mmHg, albumin in g/L and phosphate in mmol/L) [17]. The SIDa to SIDe difference should equal zero unless unmeasured charges are present in the blood. These charges are captured by the strong ion gap (SIG = SIDa - SIDe). A positive SIG value represents unmeasured anions that are needed to account for the measured pH, measured levels of strong ion and weak acids, and to assure iso-electricity [18]. Statistical analysis was performed using SPSS version 20 for Windows (SPSS Inc., Chicago, IL, USA). Chi-square and Fisher exact test were used to compare categorical variables between groups. The Mann-Whitney *U* test was applied for comparison of non-normally distributed parameters and the Wilcoxon test was used for comparing variables within a group. Values were expressed as medians (range) unless indicated otherwise.

**Table 2:** Composition of citrate and bicarbonate-buffered solutions including their calculated SIDa

Components (values in mmol/L)	Prismocitrate 10/2	Prismocitrate 18	Prismasol 2	Prismocal B22
Citrate	10	18		
Citric acid	2			
Sodium (Na+)	136	140	140	140
Chloride (Cl-)	106	86	111.5	120.5
Calcium (Ca2+)			1.75	0
Magnesium (Mg2+)			0.5	0.75
Lactate			3	3
Hydrogen carbonate (HCO3-)			32	22
Potassium (K+)			2	4
Glucose			6.1	6.1
SIDa	30	54	29.75	21.25

Values for citrate, citric acid,electrolytes, lactate and SIDa are expressed in mmol/L

## Results

Twenty-eight patients were enrolled in the PC10/2 group and 31 patients in the PC18 group. Treatment groups did not significantly differ for age, gender, type of disease, disease severity, presence of shock, and duration of mechanical ventilation. The evolution of relevant acid-base variables and electrolyte levels during the study are displayed in Table 3. Pre-treatment pH and chloride levels were comparable between groups ([7.26 (7.12–7.40) vs. 7.33 (7.22–7.44); *p* = 0.095] and [105 (99–112) vs. 106 (99–113) mmol/L; *p* = 0.326], PC10/2 vs. PC18). After 24 h of treatment, pH remained low in the PC10/2 group whereas it normalized in the PC18 group [7.28 (7.22–7.34) vs. 7.42 (7.37–7.47); *p* = 0.0001]. PC10/2 patients had higher baseline lactate concentrations. Thereafter, lactate equally decreased in both groups [from 4.6 (0–10.3) to 3.1 (0.2 – 6); *p* > 0.05 in PC10/2 patients and from 2.5 (0–5) to 2.1 (0.3–3.9); *p* > 0.05 in PC18 patients]. Lactate levels between groups were not anymore significantly different at 24 h.

**Table 3:** Relevant acid base variables and electrolytes at baseline (T0) and after 24 h (T24)

Variables	Prismocitrate 10/2	Prismocitrate 18	*P* value
pH T0	7,26 (7,12 – 7,40)	7,33 (7,22 – 7,44)	0.095
pH T24	7,28 (7,22 – 7,34)	7,42 (7,37 – 7,47)	0.0001
Na + T0	138 (133–144)	142 (134–149)	0.042
Na + T24	136 (133–139)	139 (135–145)	0.006
K+ T0	4,5 (3,7 – 5,3)	4,4 (3,7 – 5,3)	0.873
K+ T24	3,8 (3,4 – 4,2)	3,8 (3,4 – 4,2)	0.632
Cl- T0	105 (99–112)	106 (99–113)	0.326
Cl- T24	105 (102–108)	100 (95–105)	0.0001
Ca2+ T0	7,3 (6,4 – 7,2)	7,4 (6,6 – 8,2)	0.611
Ca2+ T24	8,6 (7,9 – 9,3)	9,2 (8,3 – 10,1)	0.004
Mg2+ T0	2 (1,6 – 2,4)	2,3 (1,8 – 2,8)	0.01
Mg2+ T24	1,8 (1,4 – 2,2)	2 (1,7 – 2,3)	0.040
SIDa T0	43 (38–48)	47 (41–53)	0.003
SIDe T0	27 (18–36)	39 (31–47)	0.0001
SIG T0	17 (11–23)	10 (6–16)	0.0001
SIDa T24	43 (40–43)	51 (47–55)	0.0001
SIDe T24	24 (19–29)	39 (35–43)	0.0001
SIG T24	17 (13–21)	12 (8–16)	0.0001
Lactate T0	4,6 (0–10,3)	2,5 (0–5)	0,031
Lactate T24	3,1 (0,2 – 6)	2,1 (0,3–3,9)	0,140

Values for electrolytes, SIDa, SIDe, SIG, and lactate are expressed as medians (range) in mmol/L

Calculated SIDa values between groups were significantly different at baseline [43 (38–48) vs. 47 (41–53) mmol/L; *p* = 0.003]. After 24 h, SIDa had increased in PC18 patients [from 47 (41–53) to 51 (47–55) mmol/L; *p* = 0.001] but remained unchanged in the PC10/2 group [from 43 (38–48) to 43 (40–43); *p* > 0.05], resulting in a significantly higher SIDa in PC18 as compared with PC10/2 subjects (43 vs. 51 mmol/L; *p* = 0.001). At initiation of therapy, sodium concentration was lower in the PC10/2 than in the PC18 group [138 (133–144) vs. 142 (134–149) mmol/L; *p* = 0.04]. After 24 h, sodium concentration decreased in both groups, remaining significantly lower in the PC10/2 group as compared with the PC18 group [136 (133–139) vs. 139 (135–145) mmol/L; *p* = 0.006]. Calculated citrate flow was not statistically different between the two treatment periods (2.9 vs. 3 mmol/L of blood accessing the filter; *p* > 0.05). However, when differences in chloride concentration (106 vs. 86 mmol/L) and chloride flow (9.9 vs 5.4 mmol/L of blood accessing the filter) between the two citrate formulations were related to variations in citrate volume given between the two periods (2200 vs.1500 mL/h), the chloride flow per liter blood accessing the filter was found to be significantly higher in the PC10/2 group (25.9 vs. 14.3 mmol/L blood; PC10/2 vs. PC18; *p* < 0.05). This accounted for the difference in chloride levels between groups after 24 h of treatment [105 (102–108) vs.100 (95–105) mmol/L, PC10/2 vs. PC18; *p* = 0.0001]. SIG at 24 h also remained higher in the PC10/2 group (17 vs.12 mmol/L, PC10/2 vs. PC18; *p* = 0.001). At the end of study, none of the PC10/2 patients reached a pH >7.5 but 25 % had a SIDa > 45 mmol/L. In the PC18 group, 10.3 % of the patients had pH values > 7.5 whereas 93 % were diagnosed with a SIDa > 45 mmol/L.

## Discussion

The buffering capacity of a citrate solution depends on the conversion of trisodium citrate to citric acid (Na_3_citrate + 3H_2_CO_3_ → citric acid (C_6_H_8_O_7_) + 3NaHCO_3_) and thus to the proportion of sodium as a cation. Hence, 1 mmoL trisodium citrate provides the same buffer strength as 3 mmol sodium bicarbonate, assuming that citrate is completely metabolized. Citric acid does not act as a buffer [4, 8, 18]. Following citrate metabolism, the remaining sodium increases the SIDa. Increasing SIDa (eg by infusing Plasmalyte®) produces alkalosis while the administration of a zero-SIDa solution (eg NaCl 0.9 %) will decrease SIDa and contribute to metabolic acidosis. Our findings suggest that a higher chloride-containing citrate solution (PC10/2) for RCA-CVVH may significantly reduce alkalosis-buffering capacity. One would expect that administration of PC10/2 (30 mmol/L of buffer equivalent) should be associated with a metabolic acidosis. Looking from a pure buffer equivalence perspective, this acidosis is considered to be primarily determined by a decrease in SIDa [10, 11]. Accordingly, PC18 (54 mmoL of buffer equivalent) should lead to more rapid correction of acidosis and progressive development of metabolic alkalosis. Before the start of CVVH, both patient groups had mild metabolic acidosis. This acidosis resulted from increased unmeasured anions (high SIG) in the presence of an increased lactate level and was more pronounced in the patients receiving PC10/2 [19–21]. Within 24 h, acidosis was completely reversed in all patients who received the solution with a higher citrate concentration (PC18) along with a decrease in serum chloride and an increase in SIDa. In contrast, patients who received PC10/2, developed hyperchloremic acidosis. SIDa and SIG in this group remained unchanged which counteracted citrate-induced metabolic alkalosis. Since citrate flow entering the filter was similar during the two study periods (the difference in citrate concentrations being compensated by variations in citrate volume), the observed largely uncorrected acidosis and higher SIG at 24 h in the PC10/2 group is most plausibly explained by the higher chloride content of the PC10/2 solution. The lower bicarbonate concentration in the substitution fluid administered in PC18 as compared with PC10/2 subjects (22 vs. 32 mmol/L) cannot account for the less rapid correction of acidosis. Rather, this low bicarbonate load should have attenuated late rebound alkalosis. Baseline lactate was higher in the PC10/2 group. However, lactate levels had decreased in both PC10/2 and PC18 patients at 24 h. At that time, concentrations were no longer different between groups making it unlikely that lactate significantly contributed to the persisting metabolic acidosis in patients receiving PC10/2. This leads to hypothesize that the high chloride-containing citrate solution used for RCA-CVVH significantly reduces alkalosis-buffering capacity and thus blunts correction of acidosis. This “blunting effect” may be explained by differences in chloride flow between the applied citrate solutions inducing hyperchloremic acidosis.

Our findings argue against currently used therapeutic approaches. To date, clinicians try to correct metabolic acidosis during CVVH by administering additional bicarbonate infusion [17, 22]. However, excess intravenous bicarbonate may cause unwarranted side-effects and even increase mortality [22]. Consequently, new citrate formulations have been implemented to avoid rebound metabolic alkalosis whilst assuring timely correction of acidosis and obviating the need to infuse intravenous bicarbonate. Our study underscores a potential role of the “forgotten” chloride anion in acid-base equilibrium [11, 18, 20]. Different citrate formulations, albeit infused at similar flow rates and sharing equivalent citrate-related buffer strength, may exhibit divergent capacity and speed to correct acidosis because of a substantial difference in chloride content. The lower SIDa and the higher SIG after 24 h of CVVH in patients treated with PC10/2 as compared with PC18 were related to a higher plasma chloride concentration. Interestingly, this untoward “side-effect” associated with citrate formulations has been alluded to [22–26] but was never studied in depth. Tolwani et al. compared a 0.67 % with a 0.5 % trisodium citrate replacement solution for continuous veno-venous hemodiafiltration (CVVHDF). The 0.5 % citrate solution (18 mmol/L citrate) maintained an appropriate acid-base balance whereas the 0.67 % solution (23 mmol/L citrate) resulted in a mild but unexplained alkalosis [17]. Citrate and chloride flow were comparable between groups and citrate concentration likely similar as both filter lifespan and post-filter ionized calcium were not different [17]. We suggest that applying the Stewart-Figge principle instead of a strictly pH-directed approach allows to unravel this intriguing metabolic issue. The 0.5 and 0.65 % citrate solutions used by Tolwani et al. [17] had a SID of respectively 54 and 69 mmol/L (ie equivalent of bicarbonate generation). Knowing that both solutions contained 140 mmol/L sodium, the principle of solution electro-neutrality requires chloride levels of respectively 89 and 74 mmol/L [18]. Thus, notwithstanding the use of a chloride-rich dialysate (118.5 mmol/L), the significantly lower chloride content of the 0.65 % citrate liquid induced metabolic alkalosis. Our study results, although obtained under different CRRT conditions, do corroborate these findings.

Egi et al. found that increasing the citrate dose during RCA-CVVH significantly attenuated magnitude and duration of metabolic acidosis [9]. When administering respectively 11 and 14 mmol/L citrate (ie a SIDa of respectively 33 and 42 mmol/L), they observed an alkalinizing effect depending on the SIDa of the replacement fluid and an acidifying effect due to an increase in unmeasured anions [9, 17]. The highly different citrate flow between groups (1.83 vs. 3.10 mmol/L blood, 22 vs. 28 mmol/h, and 520 vs. 672 mmol/day) might explain the observed alkalinizing effect [9]. Compared with our patients, hyperchloremic metabolic acidosis did not occur as the difference in chloride flow was less pronounced (18 vs.16.5 mmol/L blood) for a chloride content of 108 vs. 99 mmol /L [9, 11]. Interestingly, the recent observation that hypochloremic dialysis can correct metabolic acidosis by reducing unmeasured anions indirectly adds support to our findings [27].

Some limitations of our study must be recognized. Its observational and unblinded “before-after” design without randomizing patients towards a specific treatment may have rendered comparisons between groups less accurate. A possible effect of the different bicarbonate post-dilution solutions with different SIDs was not integrated in global acid-base evaluation. However, this probably had marginal importance since, unlike our findings, it should have improved acidosis in the patients receiving the lower citrate concentration [11]. As the PC10/2 formulation was initiated within the context of a newly implemented citrate protocol, an inherent learning process might have biased our results. Still, data were obtained during 24 h of treatment and an appropriate control group was available. It is unclear whether any back-diffusion of chloride [28] during CVVH occurred. Finally, one might argue that different results might be obtained with CVVHDF. However, it is doubtful that CVVHDF would have obviated hyperchloremia as chloride was provided continuously [28, 29]. Apart from being more labor-intensive and expensive [28, 30], CVVHDF also provides no superior control of electrolyte balance [31] and eventual competition between convection and diffusion at the inner part of the membrane may blunt diffusion capacity [32].

## Conclusions

In conclusion, the Stewart-Figge approach allowed to elaborate previous experience showing that metabolic acidosis is attenuated and buffer capacity increased when a citrate solution that contains less chloride is used for RCA. We postulate that a greater divergence in chloride flow accounts for the significant difference in severity and duration of metabolic acidosis observed in patients undergoing CVVH who receive different citrate solutions at similar flow rate and with equal buffer capacity. Our findings, albeit provocative and suggesting a change in attitude towards a more optimal metabolic control during RCA-CVVH, remain hypothetical and definitely need confirmation by a large randomized controlled trial.

## Bullet Points

1. RCA-CRRT in patients with AKI and metabolic acidosis should be performed with Prismocitrate 18.
2. Metabolic alkalosis can be corrected by switching to Prismocitrate 10/2 or even better by infusing natrium chloride 0.9 % instead of bicarbonate solution as substitution fluid.
3. The Stewart-Figge method may represent a better tool to assess changes in acid-base metabolism during RCA-CRRT.
4. A divergence in chloride but not citrate flow may be a plausible explanation for the difference in severity and duration of metabolic acidosis.

## Abbreviations

AKI: Acute kidney injury
AN: Acrylonitrile
CRRT: Continuous renal replacement therapy
CVVH: Continuous veno-venous hemofiltration
CVVHDF: Continuous veno-venous hemodiafiltration
RCA: Regional citrate anticoagulation
SIDa: Apparent strong ion difference
SIDe: Effective strong ion difference
SIG: Strong ion gap
ST: Surface treated
