# Correction to: Chloride content of solutions used for regional citrate anticoagulation might be responsible for blunting correction of metabolic acidosis during continuous venovenous hemofiltration

**DOI:** 10.1186/s12882-020-01749-1

**Published:** 2020-03-24

**Authors:** Rita Jacobs, Patrick M. Honore, Marc Diltoer, Herbert D. Spapen

**Affiliations:** Intensive Care Department, Universitair Ziekenhuis Brussel, Vrije Universiteit Brussel, 1090 Brussels, Belgium

**Correction to: BMC Nephrol**

**https://doi.org/10.1186/s12882-016-0334-3**

Following publication of the original article [1], we have been notified that the approved number by the Ethical Committee was given incorrectly. In the section “Methods” stated that: The Central Ethical Committee of the University Hospital approved the study protocol (B.U.N. 143201318818), this number is incorrect and should be expressed as follows: B.U.N. 143201318819.

## References

[1] Jacobs (2016). Chloride content of solutions used for regional citrate anticoagulation might be responsible for blunting correction of metabolic acidosis during continuous venovenous hemofiltration. BMC Nephrol.

